# Inflamed Amyand’s Hernia in an Elderly Male: A Rare Surgical Surprise

**DOI:** 10.7759/cureus.100131

**Published:** 2025-12-26

**Authors:** Gulalai Khan, Aysha Niaz, Maria Adan, Fatima Suleman, Imran Khan, Amit K Misra

**Affiliations:** 1 General Surgery, Swat Medical College, Swat, PAK; 2 General Surgery, Saidu Teaching Hospital, Swat, PAK; 3 Hospital Medicine, Penn State Health Holy Spirit Medical Center, Camp Hill, USA

**Keywords:** amyand's hernia, inguinal hernia surgery, intraoperative finding, rare inguinal hernia, strangulated inguinal hernia

## Abstract

Amyand's hernia is a rare anatomic occurrence, and appendicitis in Amyand’s hernia makes it exceptionally rare. We present such a rare case that was unexpectedly found during right inguinal hernia repair, highlighting the rarity and challenges in diagnosing this condition preoperatively, as well as the importance of awareness and proper management of this rare case. A 68-year-old male presented with right inguinal pain, tenderness, and swelling in the right inguinal region, initially admitted for right inguinal hernia repair. Surgery was postponed due to poor glycemic control. The patient was subsequently admitted for said surgery. During the procedure, it was found that the hernial sac contained a part of the bowel and an inflamed appendix, confirming Amyand's. The patient underwent right groin exploration, appendectomy, and hernioplasty. Through this case, we highlight the importance of intraoperative vigilance and adaptability in managing rare surgical findings, emphasizing the need for surgeons to be prepared for unexpected situations during hernia repairs.

## Introduction

Amyand's hernia is a rare type of inguinal hernia in which the appendix is found within the hernia sac (<1% incidence) [1]. Claudius Amyand, while operating on an 11-year-old boy presenting with right inguinal pain on December 6, 1735, found a stone-encrusted pin within the appendix, and the appendix itself was found in the hernial sac; thus, this condition is named after him [2]. The appendix is found in the inguinal hernia sac in less than 1% of all inguinal hernia patients. Amyand’s hernia, complicated by abscess formation or inflammation, is even rarer, with an incidence rate of 0.1% [3-4]. Most patients have no symptoms and are diagnosed during the surgery. While imaging is the key mode for diagnosis, it is a challenging diagnosis to make that requires a high index of suspicion [5]. The incidence of Amyand’s hernia as documented in the literature is estimated to occur in 0.19-1.7% of all hernia cases [6]. Since the process vaginalis is patent in children, they are three times more likely to be diagnosed with Amyand’s hernia compared to adults [7]. Appendicitis reported in an inguinal hernia is exceptionally rare, with reported incidence varying from 0.07% to 0.13% [8], while perforation of the appendix incarcerated within an inguinal hernia has a reported incidence of 0.1% of all appendicitis cases [9]. Even though rare, mortality associated with Amyand’s hernia has a documented occurrence of 14-30%, mostly due to sepsis. Another study reported a mortality rate of 5.5% in patients with early intervention and appropriate post-operative care [10-11]. 

Although the exact pathophysiology of appendicitis in Amyand’s hernia is not completely understood, it is believed that, unlike most appendicitis, where there is an intraluminal obstruction, in this case, extraluminal compression of the appendix inside the hernia leads to ischemia, bacterial overgrowth, and inflammation within the appendix [12]. We report a case that is among the 0.1% of hernia cases, an inflamed appendix found in an inguinal hernia.

## Case presentation

A 68-year-old male with a history of diabetes mellitus presented to the Outpatient Department (OPD) with the complaint of a painful swelling in the right inguinal region, ongoing for eight days. There was a positive history of weightlifting and obesity. There was no history of trauma, recent abdominal surgery, chronic cough, no recent weight loss or night sweats. The patient reported a gradual onset of pain, which was intermittent in nature, with each episode progressively worsening compared to the previous episodes, dull in character, and reaching up to 7 out of 10 on the severity scale. The pain radiated to the scrotum and lower abdomen. It was aggravated by coughing and straining and relieved with manual reduction. The symptoms were associated with nausea, intermittent constipation, but overall, being able to pass stools and flatus. No urinary symptoms were reported. The patient had a known history of diabetes mellitus with poor compliance. There was no history of any prior surgical procedures.

On physical examination, the patient was afebrile with the following vitals: blood pressure = 130/90 mmHg, pulse = 79 bpm, respiratory rate = 20/min, and temperature = 99°F.

Regional examination revealed right inguinal swelling measuring approximately 3.4 cm at the largest diameter, which was not reducible. It was tender and painful. It had a positive cough impulse, and the overlying skin was smooth with no signs of redness or ulceration. Abdominal examination showed a soft abdomen, which was non-tender and non-distended with no scar or bruising. There was mild tenderness in the hypogastric region extending to the right iliac region. The rest of the abdominal exam was normal.

The laboratory investigation findings of the patient are presented in Table 1.

**Table 1:** Laboratory findings of the patient.

Test category	Parameter	Result	Reference range
Full blood count (FBC)	Hemoglobin (Hb)	14.5 g/dL	Male: 13.5–17.5 g/dL / Female: 12–16 g/dL
	Total leukocyte count (TLC)	12.3 × 10⁹/L	4–11 × 10⁹/L
	Platelets	280,000/µL	150,000–450,000/µL
Random blood sugar (RBS)	Glucose	359 mg/dL	< 140 mg/dL (normal)
Hepatitis / HIV screen	HBsAg	Negative	Negative
HCV screen	HCV antibodies	Negative	Negative
HIV screen	HIV	Negative	Negative
Liver function tests (LFTs)	ALT	35 U/L	7–56 U/L
	Total bilirubin	0.1 mg/dL	0.1–1.2 mg/dL
Renal function tests (RFTs)	Urea (BUN)	25 mg/dL	7–20 mg/dL
	Creatinine	0.9 mg/dL	0.6–1.3 mg/dL
Coagulation profile	PT	13 sec	11–13.5 sec
	INR	1.0	0.8–1.2
	APTT	25 sec	25–35 sec

Chest X-ray and electrocardiogram did not show any acute abnormal findings.

Preoperative findings

Based on the clinical findings and ultrasound, a diagnosis of right inguinal hernia was made. Considering that the patient had ongoing pain and the hernia was irreducible, it was decided to take the patient to the OR for exploration for possible early inguinal hernia incarceration. However, considering that the patient had hyperglycemia with a blood glucose level of 359 mg/dL, surgery was postponed until the blood glucose level came down to less than 200 mg/dL. During this time, the patient received 1 L bolus of normal saline and a one-time dose of 12 units of insulin aspart subcutaneously. Two hours after the insulin administration, blood glucose levels were 186 mg/dL, and then the patient was taken to the OR for surgery. 

Procedures performed

The procedures performed were right groin exploration, appendectomy, and mesh hernioplasty.

Intraoperative findings

**Figure 1:**
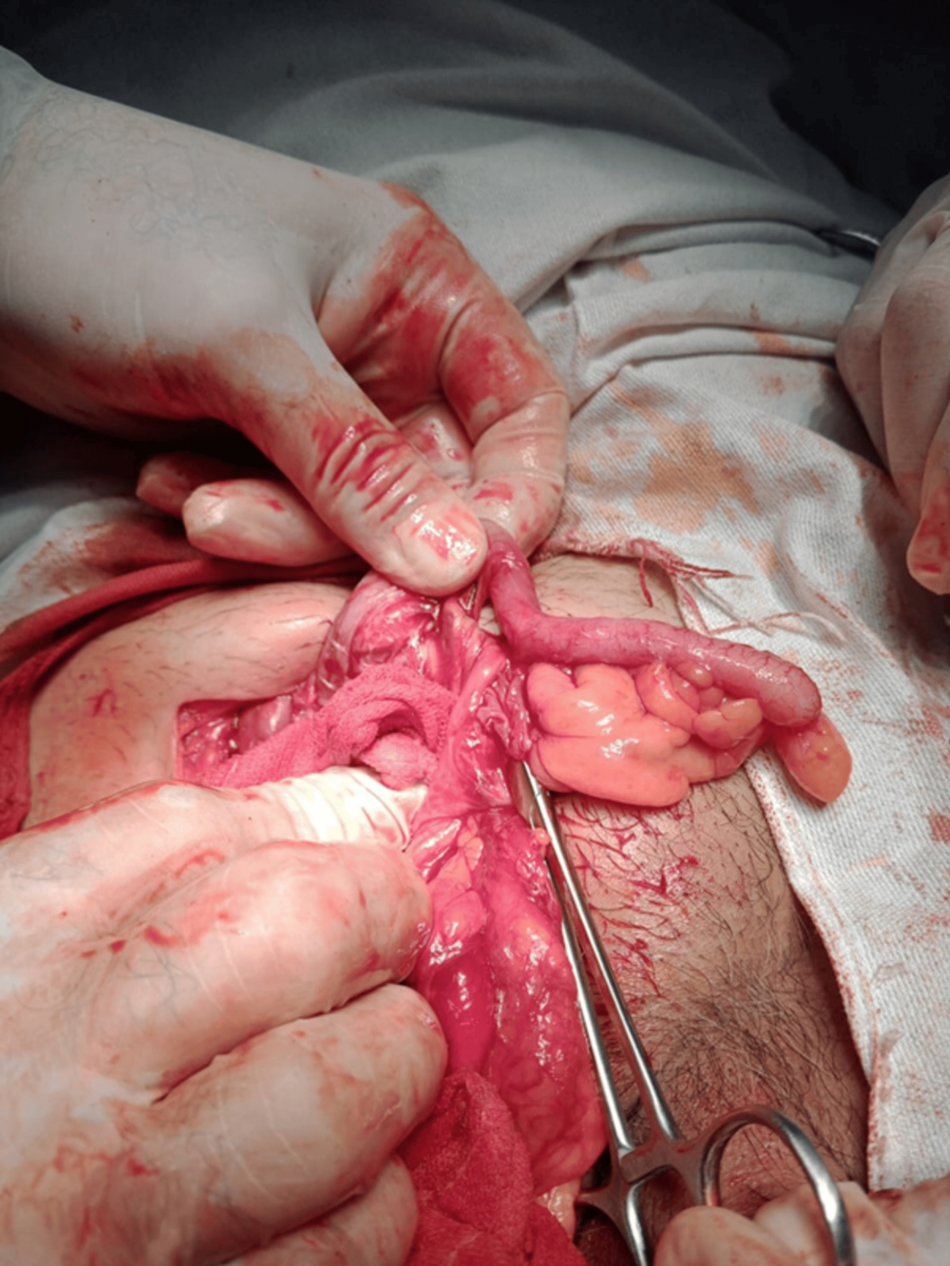
Intraoperative photograph showing the inflamed appendix within the inguinal hernia sac along with adjacent small bowel loops.

Intraoperatively, a 3 cm right inguinal hernia defect was identified. The hernial sac contained viable small bowel loops and a non-perforated, inflamed vermiform appendix, with dense adhesions between the contents and the sac wall. The appendix, as retrieved from the opened hernial sac, is shown in Figure 1.

Surgical management

After placement of the patient in the supine position and administration of general anesthesia under standard aseptic and antiseptic measures, the right inguinal region was thoroughly examined. A transverse skin incision was then made over the site. The Hernial sac was identified and isolated. Upon opening, the contents revealed the small bowel and the appendix, confirming the diagnosis of Amyand's hernia. After adhesiolysis, the small bowel was thoroughly examined to ensure viability and was found to be healthy. The appendix appeared inflamed, consistent with appendicitis. Appendectomy was performed using the standard technique. The bowel was returned to the abdominal cavity, followed by repair of the hernia with mesh hernioplasty. Layer-by-layer closure was performed, and once hemostasis was ensured, a clean antiseptic dressing was applied.

Postoperative course

The postoperative course was uncomplicated. The patient had post-op labs that were stable, and diet was advanced until he was able to tolerate a regular diet. After completing the peri-op antibiotics course, the patient was discharged home a day after surgery with two weeks of initial post-op outpatient follow-up.

## Discussion

Our patient was found to have acute appendicitis within an inguinal hernia without any involvement of contamination in the rest of the abdomen, representing type 2 Amyand's hernia according to the Losanoff-Basson classification system [13]. This classification is a generally accepted treatment algorithm for Amyand's hernia based on appendicular pathology and the presence of sepsis. Type 2 Amyand's hernia is more prevalent in children due to a patent processus vaginalis, but our case is unique as we found it in an adult patient.

The typical inguinal can vary depending on size, reducibility, and the presence or absence of complications like strangulation or incarceration. The symptoms and signs include a visible or palpable groin lump or swelling, which can be tender/non-tender, reducible/irreducible, pain can be dull or sharp depending on the severity, a positive/negative cough impulse, which can show if the swelling is connected to the abdominal cavity or not, and there will be heaviness or pressure in the groin area. The preoperative diagnosis of an Amyand's hernia is challenging because it does not have any specific signs, and most patients will present with the same signs and symptoms of any inguinal hernia. There ultrasound study by a skillful operator/interpreter or a computed tomography (CT) scan is performed. Most diagnoses are made intraoperatively [14-15].

According to Losanoff's classification, for type 2 Amyand's hernia, like our patient, a mesh should not be used after appendectomy is performed due to an increased risk of infection [13]. However, Kose et al. reported favorable outcomes with appendectomy and mesh inguinal hernia repair in cases of Amyand’s hernia with non-inflamed appendices, demonstrating that mesh placement can be safe in a clean operative field. Although their series focused on type 1 disease, their findings challenge the traditional avoidance of mesh after appendectomy and support a more individualized approach to hernia repair when contamination is minimal [16].

However, in this case, the risk of hernia recurrence was higher because he was older, obese, and worked in a labor-intensive job. Therefore, following appendectomy and confirmation of a clean operative field, a Lichtenstein tension-free hernioplasty was performed with a successful outcome. The mesh was placed to reinforce the posterior wall of the inguinal canal, providing durable repair without tissue tension and reducing the risk of recurrence. This approach is supported by recent literature. Velimezis et al. reported no recurrence or infection at 36-month follow-up after a successful mesh repair in a 78-year-old with recurrent type 2 Amyand's hernia [17]. Favorable outcomes were also demonstrated by Ali et. al over 1-3 years in 3 cases managed similarly [18]. Thus, we recommend an individualized approach. In cases where there is appendicitis without perforation, depending on the overall health and hernia size, hernia repair with a mesh may be a good option, as the risk of the occurrence of infection is lower compared to the lifelong risk of recurrence of hernia with primary hernia repair. However, significant debate persists over the widely accepted classification system and management strategies, and additional evidence-based research is needed for future optimization of patient outcomes [19].

## Conclusions

Amyand’s hernia with appendicitis is a rare surgical condition but carries significant mortality that can be significantly reduced with timely and appropriate intervention. This case of type 2 Amyand's hernia emphasizes the need for vigilance during inguinal hernia surgeries, where the surgeon needs a keen eye to find the appendix with or without appendicitis in the inguinal hernia sac. Widely accepted recommendations exclude mesh use in cases where the field may be contaminated; however, we felt our patient was a good candidate for a mesh repair, as the risk of recurrence was high due to obesity, old age, and a labor-intensive job. Evolving evidence also favors a more individualized approach based on contamination level and other patient-specific factors, thus supporting our individualized approach. Further prospective research is required to establish evidence-based guidelines for optimal management of this rare surgical entity.
